# End-to-End Pipeline Integrating Local Small Language Models and Machine Learning for Data Extraction and Stroke Outcome Prediction in Emergency Department

**DOI:** 10.34133/csbj.0064

**Published:** 2026-04-30

**Authors:** Junsu Kim, Ji Hoon Kim, Arom Choi

**Affiliations:** ^1^ Department of Emergency Medicine, Yonsei University College of Medicine, Seoul 03722, Republic of Korea.; ^2^ Yonsei Institute for Digital Healthcare, Yonsei University, Seoul 03722, Republic of Korea.

## Abstract

**Background:** A substantial proportion of clinical data is stored in unstructured text, limiting its utility for evidence generation. Small language models (SLMs) can extract information but face hallucination risks and privacy concerns. Locally deployable SLMs are needed for secure and reliable healthcare use. **Methods:** We designed an end-to-end pipeline integrating clinical text extraction with stroke outcome prediction. Records of 1,398 patients screened for ischemic stroke were reviewed, with 1,166 included. A Llama 3 8B SLM was fine-tuned using low-rank adaptation and 4-bit quantization for local feasibility, guided by few-shot prompting. Multi-tiered validation—rule-based checks, retrieval-augmented generation, cosine similarity flagging, and human-in-the-loop review—was implemented. Structured data from 767 patients were used to train models predicting poor outcomes at 3 months. **Results:** Baseline extraction accuracy was 64.9% (95% CI, 62.0% to 67.8%), improving to 86.0% after fully automated multi-tiered validation, and further to 97.0% (95% CI, 95.7% to 98.3%) following human-in-the-loop review. Template-based variables achieved F1 > 0.90 (95% CI, 0.88 to 0.96). Narrative extraction reached F1 = 0.87 (95% CI, 0.84 to 0.90). NIHSS scores were extracted with a mean absolute error of 0.853 (95% CI, 0.791 to 0.915). TabPFN achieved an AUROC of 0.816 (95% CI, 0.784 to 0.847) with good calibration, confirming reliable risk stratification. **Conclusion:** This study demonstrates a privacy-preserving, efficient pipeline for clinical text processing. By combining an SLM with multi-tiered validation and predictive modeling, it offers a proof-of-concept solution with potential for broader deployment to transform unstructured records into structured data suitable for stroke outcome research and decision-support modeling.

## Highlights

- Local small language model achieves 97.0% extraction accuracy through multi-tiered validation
- Privacy-preserving pipeline processes clinical text without cloud infrastructure
- Automated extraction enables stroke outcome prediction with AUROC 0.816
- Operationally feasible inference (8.3 s) supports clinical deployment feasibility

## Introduction

The digital transformation of healthcare has led to an exponential growth of electronic health records (EHRs), creating unprecedented opportunities for data-driven medicine [1]. However, a significant portion of this data is captured in an unstructured narrative format, such as clinical notes, discharge summaries, and radiology reports [2,3]. This vast repository of unstructured data contains rich, nuanced clinical information essential for precision medicine and clinical research, yet it remains largely inaccessible to traditional computational methods due to its inherent noise, variability, and complexity. Efforts to manually abstract this data are not scalable, being both time-consuming and prone to inter-rater variability [2–4].

Historically, the primary approach to unlocking these data has been through traditional natural language processing (NLP) techniques, including rule-based systems utilizing regular expressions, and classical machine learning models [5]. While these methods have shown utility in specific, narrow tasks, they often suffer from significant limitations. They typically require extensive, manually intensive feature engineering by domain experts, are expensive to develop and maintain, and lack portability across different institutions or even different departments within the same hospital due to variations in documentation practices [5]. This brittleness has been a major barrier to the widespread, scalable deployment of NLP in real-world clinical settings.

The advent of the Transformer architecture, introduced in the seminal paper “Attention is All You Need”, marked a paradigm shift in NLP [6]. Foundational models, pretrained on massive text corpora, such as BERT (bidirectional encoder representations from transformers) and the generative pretrained transformer (GPT) series, have demonstrated a remarkable ability to understand context, semantics, and nuanced relationships in text without task-specific feature engineering [7,8]. This has led to the development of state-of-the-art large language models (LLMs) that have shown extraordinary success in complex medical applications.

Recent studies have begun to evaluate the practical utility of LLMs for structured information extraction from clinical texts. A scoping review of 16 studies published between 2019 and 2025 found that GPT-4 was the top-performing model in the majority of studies, achieving greater than 85% accuracy or F1 score in clinical entity extraction tasks [9]. However, the same review highlighted substantial performance variability across document types and clinical domains, and noted that all but one study relied on real patient data transmitted to cloud-based application programming interfaces, (APIs) raising significant privacy and confidentiality concerns. Consistent with these findings, Kim et al. demonstrated that while GPT models perform adequately on straightforward extraction tasks using simple prompts, complex clinical variables requiring contextual interpretation—such as BMI (body mass index) calculation or ambiguous abbreviations—necessitate task-specific prompt engineering and continuous human oversight [10]. Importantly, several studies underscore that until privacy-preserving deployment strategies are established, the use of proprietary cloud-based LLMs on real EHR data remains ethically and regulatory constrained.

Despite these promising advances, deploying large-scale language models in healthcare is challenging due to issues of reliability and security. The phenomenon of “hallucination”—generating factually incorrect information—is a critical risk in a clinical context [11]. Furthermore, the predominant high-performance models are proprietary and accessible only through cloud-based APIs, requiring the transmission of sensitive protected health information to third-party servers. This process raises profound data privacy, security, and regulatory concerns that conflict with regulations [12].

To address this trade-off, a new approach is necessary: one that harnesses the contextual understanding of LLMs while ensuring data privacy and factual reliability. The recent proliferation of powerful, open-source small language models (SLMs), such as the Llama series [13], has created an opportunity for such a solution. These models are compact enough to be deployed and fine-tuned on local, on-premise infrastructure. In this paper, we propose and validate an end-to-end pipeline that leverages a locally deployed, fine-tuned SLM for structured data extraction from clinical notes. We detail a multi-tiered validation framework designed to maximize accuracy and minimize error. Finally, we demonstrate the utility of this automatically structured data by developing and evaluating a robust machine learning model for predicting neurological outcomes in acute stroke patients, thereby showcasing a complete, practical, and secure solution from raw text to structured data suitable for downstream research, clinical decision support, and quality improvement.

## Methods

### Study design and setting

This retrospective observational study analyzed the EHRs and brain computed tomography (CT) scans of patients with suspected ischemic stroke who were admitted to the emergency department (ED) of a single tertiary teaching hospital from July 2020 to July 2023. Patients over 18 years of age were enrolled if they had complaints of stroke-related symptoms in the last 12 h and the brain salvage through emergency stroke therapy (BEST) protocol had been activated. Patients were excluded if they had cerebral hemorrhage, newly detected intracranial tumorous lesions explaining the patient’s symptoms, or missing outcome data. The study was conducted in accordance with the tenets of the Declaration of Helsinki and was approved by the institutional review board of Yonsei University Health System (approval number 4-2025-0125). The institutional review board also waived the requirement for informed consent due to the retrospective observational design of the study.

#### BEST critical pathway protocol

The BEST protocol at the study site represents an approach to the treatment of acute ischemic stroke patients. Developed with the goal of achieving optimal treatment outcomes, this protocol employs a team approach to rapidly administer thrombolytic therapy. Its primary aim is to minimize the time elapsed between a patient’s arrival at the ED and the initiation of thrombolytic therapy, ensuring that diagnosis and intervention occur within the shortest possible time frame. Time is a critical factor, and the protocol is applicable to patients who arrive at the ED within 12 h of symptom onset, with specific provisions for those experiencing severe symptoms such as aphasia or significant limb weakness within 0 to 24 h. Activation of the protocol is determined by symptomatology, which includes sudden weakness or numbness on one side of the body, confusion, speech difficulties, visual impairment, severe unexplained headaches, and issues with walking or balance, especially when combined with other symptoms. Upon activation by an emergency physician within 10 min of arrival at the ED, the protocol involves a prompt neurological examination, initial National Institutes of Health Stroke Scale (NIHSS) calculation, CT imaging within 10 min of activation, and, for patients arriving within 4.5 h of onset, immediate intravenous (IV) tissue plasminogen activator (t-PA) administration if no cerebral hemorrhage is observed on noncontrast CT. In cases where CT angiography reveals relevant artery occlusion, IV t-PA bolus injection is followed by intra-arterial (IA) thrombectomy, provided the patient falls within the designated time window. However, if the Alberta Stroke Program Early CT (ASPECT) score is 4 points or lower, IA thrombectomy is not pursued, even within the designated time frame.

#### Dataset collection

After obtaining approval from the Data Review Board, the dataset was acquired through the Clinical Research Analysis Portal, where all patient information was fully de-identified to ensure compliance with privacy regulations. The dataset primarily consists of unstructured neurological records, including neurologists’ notes containing details on patients’ medical history, NIHSS scores, and intervention status, such as IV t-PA administration and IA procedures. The structured variables collected include demographic and clinical data such as sex, age, initial NIHSS score, hypertension, diabetes mellitus, cardiovascular disease, atrial fibrillation, dyslipidemia, prior stroke, cancer, and end-stage renal disease. Additionally, brain imaging data, including brain magnetic resonance imaging (MRI) findings interpreted by neuroradiologists and ASPECT score derived from the RAPID ASPECT scoring system, were collected.

### SLM architecture and fine-tuning

#### Model selection and hardware configuration

We selected the Llama 3 8B model as it represents a favorable trade-off between high performance and local computational requirements. Unlike larger models that require cluster-level graphics processing unit (GPU) resources, the 8B parameter model, especially when quantized, can be effectively run on a single high-end workstation, aligning with our goal of creating a locally deployable solution. All model training and inference were performed on an Apple Silicon M2 Max workstation with 64 GB unified memory and 12-core CPU/38-core GPU architecture. This consumer-grade hardware demonstrates the accessibility of our approach for resource-limited healthcare settings.

#### Text preprocessing and tokenization

Although Llama 3 8B was primarily pretrained on English text, it demonstrates substantial multilingual capabilities, including support for Korean language processing [13]. The model’s training corpus included diverse multilingual data, enabling cross-lingual transfer learning. Our domain-specific fine-tuning on institutional Korean neurology records further enhanced its ability to process Korean medical terminology and clinical documentation patterns. The SentencePiece tokenizer used by Llama 3 employs byte-pair encoding (BPE) that can effectively handle Korean characters and mixed Korean–English clinical text commonly found in our institutional records [14].

Raw clinical text from neurological records underwent several preprocessing steps. This included text normalization, expansion of common clinical abbreviations based on an institutional dictionary (e.g., “HTN” to “hypertension”, “DM” to “diabetes mellitus”, and “CVA” to “cerebrovascular accident”), and standardization of medical terms to ensure consistency. Korean–English mixed terminology was preserved as is to maintain clinical context, as standard medical abbreviations and English terms are commonly interspersed in Korean clinical documentation. Following preprocessing, the complete text was tokenized using the LLaMA SentencePiece tokenizer [14] and provided in its entirety to the model as input context.

Korean clinical notes averaged 1,247 characters (SD: 342), translating to 1,421 tokens (SD: 389)—a token-to-character ratio of 1.14, higher than English text (0.4 to 0.6) due to Korean linguistic properties and the English-centric tokenizer. All notes remained within the 4,096-token context window (maximum utilization: 58.5%). Rather than presegmenting notes, we provided full clinical narratives to the model. Relevant information—comorbidities, NIHSS scores, and interventions—was typically scattered across multiple sections interspersed with extraction-irrelevant content. This approach leverages transformer attention mechanisms to autonomously locate relevant variables within lengthy, unstructured text, simulating real-world clinical workflows where information is distributed throughout comprehensive documentation.

#### Parameter-efficient fine-tuning

To adapt the SLM to our specific clinical domain, we used low-rank adaptation (LoRA) [15]. Instead of retraining all model parameters, which is computationally prohibitive, LoRA introduces small, trainable low-rank matrices into the attention layers, drastically reducing computational cost and preventing catastrophic forgetting. We used a rank (*r*) of 16 and an alpha (α) of 32, with the AdamW optimizer and a cosine learning rate scheduler over 3 epochs. These hyperparameters were selected based on established best practices for domain adaptation of SLMs [15]. To ensure feasibility on local hardware, we applied 4-bit quantization using the bitsandbytes library, reducing the model’s memory footprint from approximately 16 GB to 4 GB while maintaining >95% of full-precision performance based on validation set metrics. The fine-tuning dataset consisted of 450 manually annotated clinical records (separate from the main study cohort) with ground-truth structured extractions verified by 2 independent clinicians. Training converged after approximately 6 h on the specified hardware. Separately, ground-truth structured annotations for all 1,166 records in the main study cohort were independently established by 2 clinicians and verified through consensus prior to any pipeline evaluation. These annotations served exclusively as the reference standard for accuracy assessment at each validation stage, ensuring complete independence between the fine-tuning and evaluation processes.

### Information extraction and validation pipeline (Fig. 1)

#### Prompt engineering

A few-shot prompting strategy was employed to guide the model through representative examples, instructing it to act as a meticulous medical data abstractor with clearly delimited input text and a predefined JSON output structure specifying data types for each variable (e.g., {“NIHSS”: “integer”, “Hypertension”: “string”}) [16]. Three examples (3-shot prompting) were included in each extraction prompt to illustrate basic patterns while intentionally excluding the full variability of real-world clinical documentation. This conservative design established a realistic baseline reflecting practical deployment conditions in which representative examples cannot capture all edge cases, documentation styles differ across clinicians and contexts, and novel phrasings or institution-specific conventions continue to emerge.

**Fig. 1.**
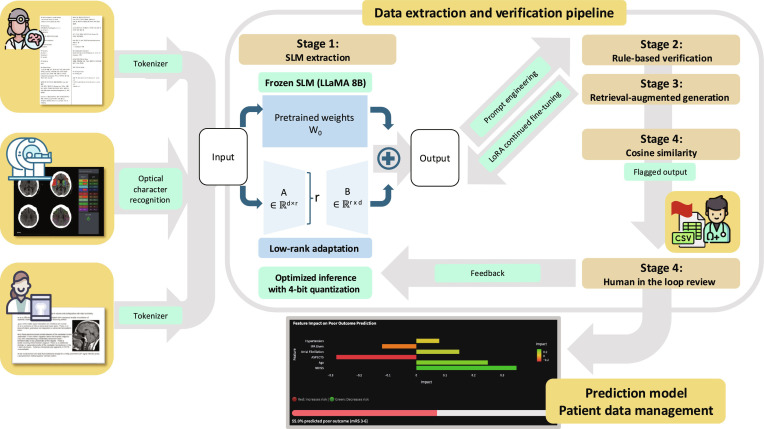
Conceptual framework of the integrated SLM-based data extraction and prediction pipeline, depicting data flow through preprocessing, SLM extraction, rule-based verification, RAG, cosine similarity flagging, HITL review, and machine learning modeling with labeled arrows.

#### Multi-tiered validation framework

The multi-tiered validation framework was designed according to a defense-in-depth principle, wherein each successive layer targets a distinct category of residual error not addressable by its predecessor: Rule-based validation addresses structural and logical formatting errors; retrieval-augmented generation (RAG)-based cross-referencing mitigates hallucinations through evidentiary grounding; cosine similarity thresholding detects semantic drift between source context and extracted output; and human-in-the-loop (HITL) review captures clinically nuanced cases requiring human judgment. This hierarchical design maximizes data fidelity while minimizing unnecessary computational and human burden, ensuring that clinician attention is directed only toward cases with the highest likelihood of error.

• Rule-based verification: This initial layer served as a rapid filter for syntactic and range errors. A set of Python scripts implemented rules such as verifying that the NIHSS score was an integer between 0 and 42, that binary variables contained only “yes”/“no”/“unknown” values, and that extracted dates fell within plausible ranges. Regular expressions were used to detect format inconsistencies in structured fields.
• RAG verification: An internal knowledge base was constructed by generating 1,024-dimensional embeddings of clinical notes and sentences using the multilingual-e5-large model and indexing them in a FAISS (Facebook AI Similarity Search) database configured with IndexFlatIP for exact inner-product similarity search, containing 1,166 document-level and approximately 45,000 sentence-level vectors [17]. For each extracted entity, a query embedding was used to retrieve the top 3 semantically similar text segments (*k* = 3) from the original note, which were concatenated and provided to the SLM in a follow-up prompt to verify or correct the extracted value: “Based on the following text from the original note: [retrieved text], verify if the extracted value [entity] is correct. If not, provide the corrected value.” If no retrieved segment exceeded a cosine similarity threshold of 0.7, the case was automatically flagged for human review rather than force-corrected by the model.
• Cosine similarity flagging: The semantic vector of the full extracted JSON object was compared against a library of 200 historical, validated records from a prior quality improvement initiative. Cosine similarity scores were calculated, and records falling below the 5th percentile (similarity < 0.82) were automatically flagged as potential outliers [18]. This threshold was derived from the cosine similarity distribution of this independent record library, which was entirely separate from the main study cohort, ensuring that threshold selection did not introduce optimistic bias into extraction or predictive performance evaluation. This step served as a supplementary safety net to catch subtle contextual anomalies not identified by previous automated steps, such as internally inconsistent combinations of variables (e.g., t-PA administration in a patient with documented contraindications). Approximately 8% of records were flagged at this stage, of which 62% were confirmed to contain errors upon human review.
• HITL review: The final validation was conducted by 2 independent clinicians—a board-certified emergency physician and a board-certified neurologist with 6 years of experience—both blinded to the model outputs. Using a stratified sampling strategy, all flagged records (identified via cosine similarity or RAG fallback mechanisms) and a random 10% sample of nonflagged records were selected for review. Reviewers compared model extractions against the original clinical notes using a standardized electronic form, marking each field as “correct”, “incorrect”, or “ambiguous”. Discrepancies between reviewers were resolved through discussion with the senior author. Confirmed errors were incorporated into continued fine-tuning of the LoRA adapters, creating an iterative feedback loop for model refinement. The remaining unflagged records were accepted as validated by the automated pipeline, establishing a hybrid human–machine validation framework.

All LoRA adapter updates triggered by HITL corrections were logged with timestamps, correction type, and responsible reviewer identity. Versioned snapshots of adapter states were maintained prior to each update cycle to enable rollback in the event of performance degradation.

### Stroke outcome prediction modeling

The pipeline follows a strict sequential architecture where SLM-based extraction is finalized prior to prediction modeling. To maintain data integrity, the SLM was fine-tuned solely for linguistic parsing of clinical narratives without exposure to downstream outcome labels (label blindness). Dataset partitioning for the prediction module (training, validation, and test sets) was performed only after the extraction stage was completed, ensuring that the predictive evaluation remains functionally independent from the feature extraction refinement.

The structured dataset was validated and used to develop models for predicting a poor neurological outcome. The dataset was divided into training (60%), validation (20%), and testing (20%) sets using a stratified split. Given the class imbalance, the synthetic minority over-sampling technique (SMOTE) was applied to the training set after feature standardization and categorical encoding to generate synthetic minority samples in the normalized feature space. Although SMOTE interpolation between binary variables may produce non-integer values, explicit rounding was not required as tabular prior-fitting network’s (TabPFN) decision tree-based architecture implicitly resolves intermediate values to binary boundaries during inference. Class-weighted loss was considered as an alternative strategy but was not implemented due to TabPFN v0.1.8’s native architecture constraints. Variables were selected including age, sex, initial NIHSS score, hypertension, diabetes mellitus, dyslipidemia, atrial fibrillation, previous cerebrovascular accident, presence of acute infarction on MRI, ASPECT score, administration of IV t-PA, and IA intervention [19,20]. The trio of models was selected to span the spectrum of complexity and interpretability: logistic regression, CatBoost, and TabPFN. Logistic regression was implemented in Python using the sklearn.linear_model.LogisticRegression package (scikit-learn v1.2.2), CatBoost was implemented using the catboost.CatBoostClassifier package (catboost v1.1.1), and TabPFN was implemented using the tabpfn.PFNClassifier package (tabpfn v0.1.8) in Python. The models were developed and trained in Python 3.10.6 using key dependencies, including NumPy (v1.23.5), Pandas (v1.5.3), Scikit-learn (v1.2.2), and Matplotlib (v3.6.2) for visualization. Model interpretation was performed using SHapley Additive exPlanations (SHAP) [21].

### Outcome measures

The primary outcome of this study was to assess the accuracy of structured data extraction by the fine-tuned SLM from unstructured clinical text. The extracted data were validated against reference standard annotations provided by experienced clinicians. The secondary outcome was the predictive performance of a machine learning model in estimating stroke outcomes based on the extracted clinical variables. The primary endpoint for outcome prediction was a poor neurological outcome based on the dichotomized modified Rankin scale (mRS), where a good outcome was defined as mRS 0 to 2 and a poor outcome as mRS 3 to 6. The mRS was assessed at 3 months post-discharge to evaluate functional outcomes.

### Statistical analysis

Continuous variables were presented as mean ± standard deviation (SD) for normally distributed data and as median with interquartile range (IQR) for non-normally distributed data. Categorical variables were expressed as counts and percentages.

To evaluate the data extraction accuracy of the fine-tuned SLM within the pipeline, the extracted data underwent a multi-step verification process. The performance of the SLM extraction was quantified by calculating precision, recall, and accuracy, each reported with 95% confidence intervals (CIs). In addition to these standard metrics, we employed several advanced evaluation methods. Variable-level F1 scores—the harmonic mean of precision and recall—were calculated to assess accuracy for each specific data field independently. To specifically measure the effectiveness of the RAG implementation, grounding accuracy was calculated, defined as the percentage of extractions correctly verified against the retrieved source documents. To evaluate the semantic similarity of narrative extractions, we utilized BERTScore, which computes a similarity score by aligning contextual embeddings, and recall-oriented understudy for Gisting evaluation (ROUGE-L), which measures the longest common subsequence between the model’s output and the reference text. To evaluate whether stepwise improvements in data extraction accuracy were statistically significant, McNemar’s test was applied to paired field-level extraction outcomes across consecutive validation stages, as each record underwent all stages sequentially. To account for multiple comparisons across validation stages (baseline versus rule-based, rule-based versus RAG, RAG versus HITL, and baseline versus HITL), Bonferroni correction was applied, with the adjusted significance threshold set at α = 0.0125 (0.05/4 comparisons).

For stroke outcome prediction, the predictive performance of the machine learning models was evaluated using the area under the receiver operating characteristic curve (AUROC) with 95% CIs, as well as area under the precision–recall curve (AUPRC) for each model. To assess model calibration, observed event probabilities were compared with predicted probabilities. Hosmer–Lemeshow test was conducted to evaluate the goodness-of-fit of the models by statistically comparing observed versus expected probabilities. All statistical analyses were conducted using Python 3.10.6 and R version 4.2.3 (www.R-project.org). A 2-sided *P* value of <0.05 was considered statistically significant in this study.

## Results

### Patient demographics

**Fig. 2.**
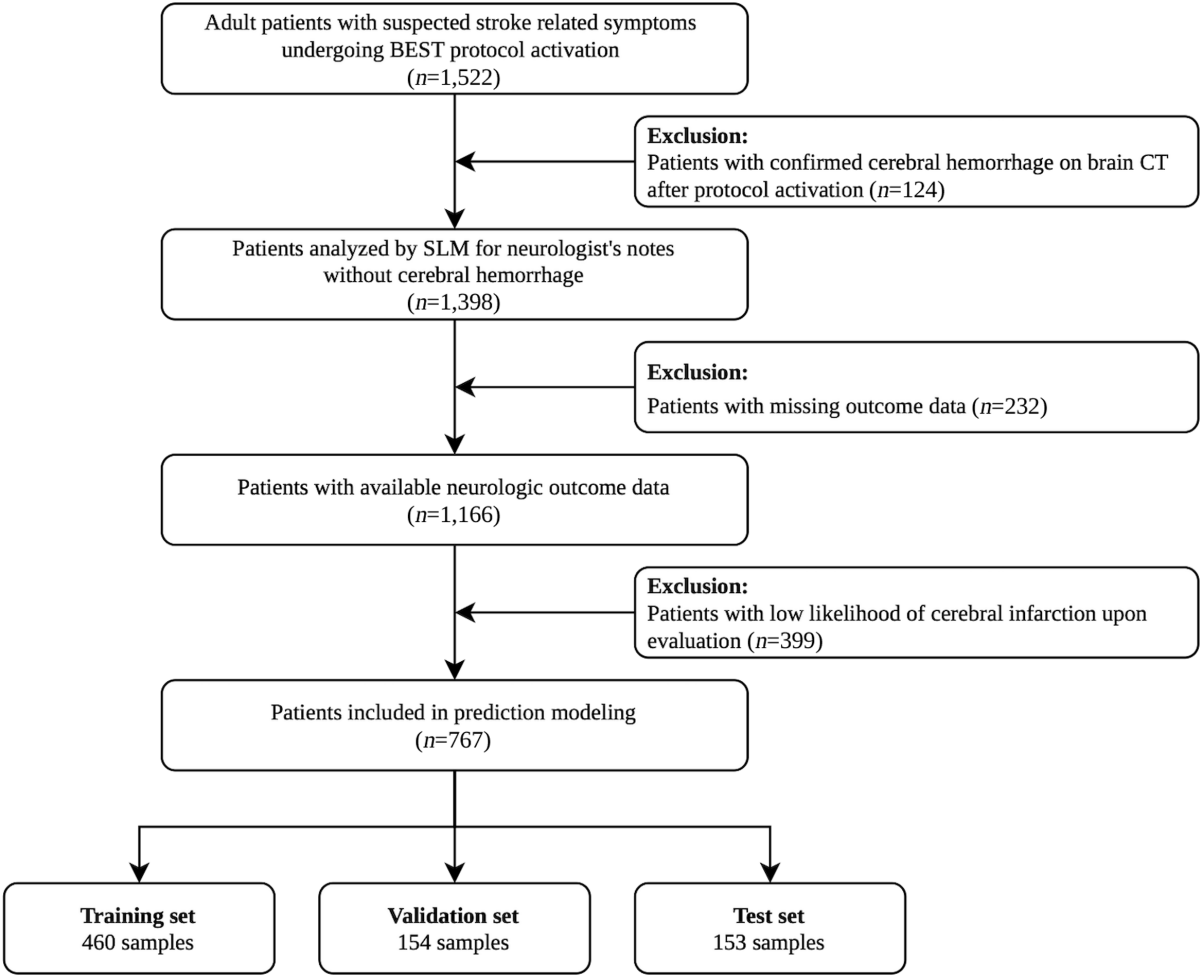
Patient flow diagram for prediction modeling, illustrating progression from 1,398 screened patients to 1,166 included and 767 used for modeling. BEST, brain salvage through emergency stroke therapy; CT, computed tomography; SLM, small language model.

The study cohort consisted of 1,166 patients, with a mean age of 65.68 ± 15.90 years; 56.2% were male. Hypertension was the most common comorbidity (57.4%), followed by diabetes (24.4%) and dyslipidemia (19.7%). The median initial NIHSS score was 3 (IQR, 1 to 7). The patient selection process for prediction modeling is detailed in Fig. 2. Detailed demographic and clinical characteristics are presented in Table 1.

**Table 1.** Patient demographics of 1,166 included patients

Variable		
Age, years (mean, SD)	65.68	15.90
Male sex (*n*, %)	655	56.2%
Past medical history		
Hypertension (*n*, %)	669	57.4%
Diabetes mellitus (*n*, %)	285	24.4%
Cardiovascular disease (*n*, %)	233	20.0%
Atrial fibrillation (*n*, %)	174	14.9%
Dyslipidemia (*n*, %)	230	19.7%
Old cerebrovascular accident (*n*, %)	253	21.7%
Malignancy (*n*, %)	134	11.5%
End-stage renal disease (*n*, %)	28	2.4%
MRI finding		
Presence of acute infarction on MRI (*n*, %)	693	59.4%
No lesion on MRI (*n*, %)	468	40.1%
Other lesions on MRI (*n*, %)	5	0.4%
Scoring		
ASPECT score (median, IQR)	9	8–10
Initial NIHSS score (median, IQR)	3	1–7
0 (*n*, %)	296	25.4%
1–4 (*n*, %)	470	40.3%
5–15 (*n*, %)	307	26.3%
16–20 (*n*, %)	66	5.7%
21–42 (*n*, %)	27	2.3%
Interventions		
Administration of IV t-PA (*n*, %)	105	9
Intra-arterial intervention (*n*, %)	87	7.5
Poor outcome at 3 months, mRS 3–6 (*n*, %)	218	28.4%

SD, standard deviation; IQR, interquartile range; MRI, magnetic resonance imaging; ASPECT, Alberta Stroke Program Early CT score; NIHSS, National Institutes of Health Stroke Scale; IV t-PA, intravenous tissue plasminogen activator; mRS, modified Rankin scale

### Data extraction performance

**Fig. 3.**
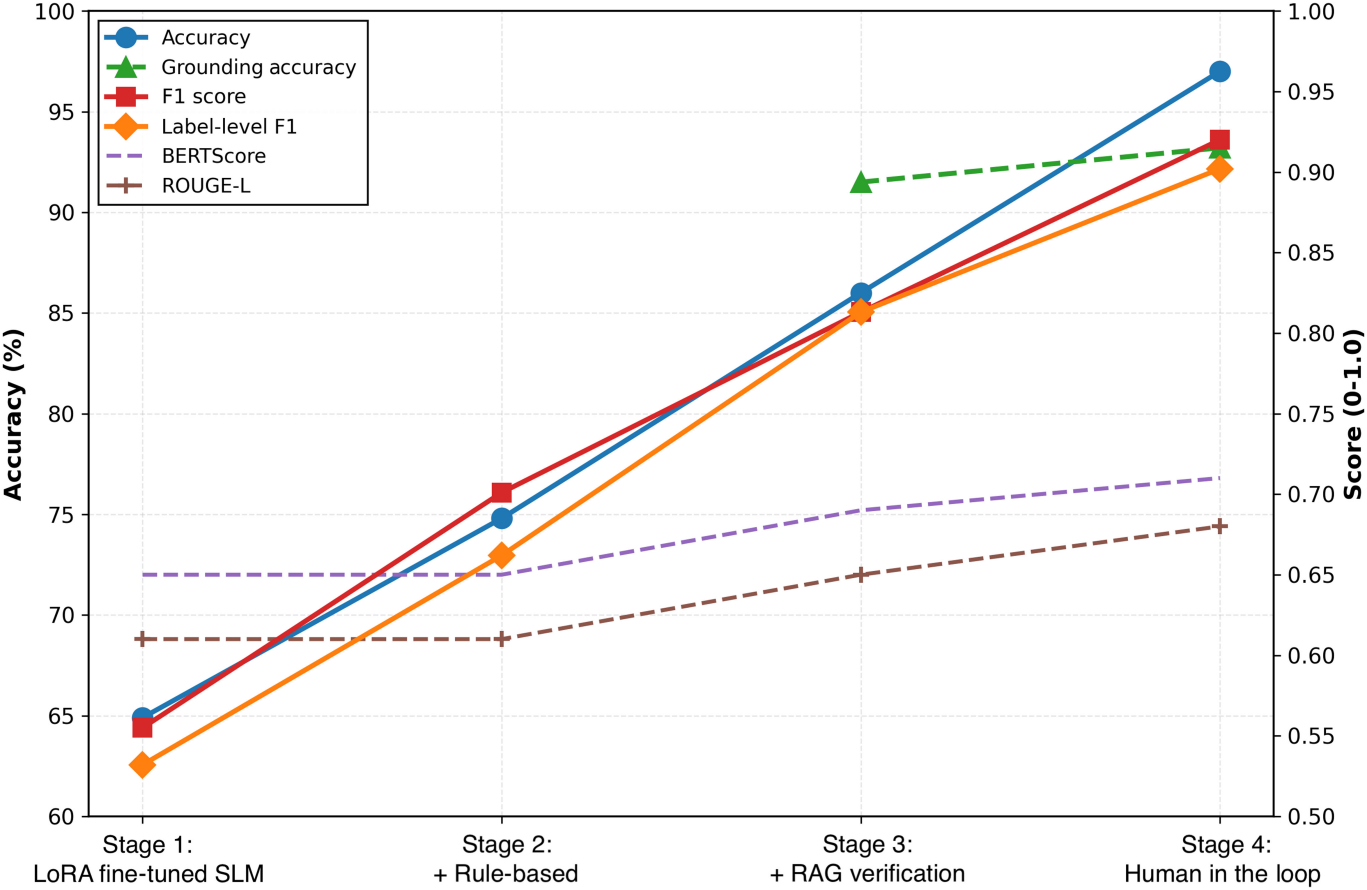
Progressive improvement in data extraction performance across validation stages. The graph shows accuracy and grounding accuracy (left axis, %) and various performance scores (right axis, 0 to 1.0) at each stage. F1 score represents micro-averaged (patient-weighted) performance across all predictions, while variable-level F1 represents macro-averaged (variable-level) performance. Grounding accuracy was introduced at the retrieval-augmented generation (RAG) verification stage and measures the percentage of extractions correctly verified against retrieved source documents. BERTScore and ROUGE-L assess semantic similarity of narrative extractions.

The effectiveness of the multi-tiered validation pipeline was assessed by evaluating performance at each sequential stage. Figure 3 illustrates the progressive improvement from the baseline few-shot SLM output to the final human-verified dataset. The initial few-shot output demonstrated an accuracy of 64.9% (95% CI, 62.0% to 67.8%), F1 score of 0.555 (95% CI, 0.524 to 0.586), and variable-level F1 of 0.532 (95% CI, 0.501 to 0.563). With the addition of rule-based verification, performance improved to an accuracy of 74.8% (95% CI, 72.1% to 77.5%), F1 score of 0.701 (95% CI, 0.670 to 0.732), and variable-level F1 of 0.662 (95% CI, 0.631 to 0.693). After RAG-based contextual validation, accuracy increased further to 86.0% (95% CI, 83.6% to 88.4%), with F1 score and variable-level F1 both 0.813 (95% CI, 0.791 to 0.835). Grounding accuracy, introduced at this stage, was 91.5% (95% CI, 89.9% to 93.1%). The final HITL review achieved the highest data fidelity, yielding an accuracy of 97.0% (95% CI, 95.7% to 98.3%), F1 score of 0.920 (95% CI, 0.879 to 0.961), and variable-level F1 of 0.902 (95% CI, 0.841 to 0.962), with grounding accuracy increasing to 93.2% (95% CI, 91.7% to 94.7%). The baseline few-shot output (stage 1), rule-based verification (stage 2), and RAG-augmented validation (stage 3) represent fully automated processing without any human intervention, while the final HITL review (stage 4) incorporates clinician oversight for flagged records. McNemar’s tests with Bonferroni correction confirmed that each successive validation stage yielded a statistically significant improvement in extraction accuracy (all *P* < 0.001, adjusted α = 0.0125), supporting the independent contribution of each pipeline component. While correctness-based metrics improved markedly, semantic similarity metrics rose more moderately: BERTScore improved from 0.65 (95% CI, 0.63 to 0.67) to 0.71 (95% CI, 0.69 to 0.73), and ROUGE-L from 0.61 (95% CI, 0.58 to 0.64) to 0.68 (95% CI, 0.65 to 0.71). Each validation component—rule-based filtering, RAG verification, and human review—contributed to statistically significant gains (*P* < 0.001 for all comparisons), confirming the additive benefit of each step in ensuring extraction fidelity.

Following the full validation pipeline, the extraction performance for each specific clinical variable was assessed, as detailed in Table 2. Values in Table 2 represent macro-averaged (variable-level) F1, whereas Fig. 3 shows micro-averaged (patient-weighted) F1 to illustrate overall performance.

**Table 2.** Post-verification extraction performance for variable types, reporting metrics (F1 score, grounding accuracy, MAE) across baseline, rule-based, RAG, and HITL stages. Values represent macro-averaged (variable-level) F1 scores. Variables are grouped by extraction type: template-based binary, narrative-based binary, and numerical. Note: Figure 3 reports micro-averaged (patient-weighted) F1 for overall pipeline performance comparison across stages. *P* values derived from McNemar’s tests with Bonferroni correction. Baseline to rule-based: *P* < 0.001, rule-based to RAG: *P* < 0.001, RAG to HITL: *P* < 0.001. Baseline to HITL: *P* < 0.001.

Variable	Metrics	Baseline (few-shot)	Rule-based	RAG	HITL
Template-based (binary)					
Hypertension	F1 score	0.568 (0.543–0.593)	0.674 (0.643–0.705)	0.813 (0.791–0.835)	0.932 (0.911–0.943)
	Grounding accuracy	–	–	91.5% (89.9–93.1%)	93.2% (91.7–94.7%)
Diabetes mellitus	F1 score	0.555 (0.526–0.584)	0.661 (0.632–0.690)	0.802 (0.781–0.823)	0.923 (0.901–0.945)
	Grounding accuracy	–	–	91.0% (89.3–92.7%)	93.0% (91.5–94.5%)
Dyslipidemia	F1 score	0.541 (0.510–0.572)	0.653 (0.621–0.685)	0.792 (0.771–0.813)	0.914 (0.892–0.936)
	Grounding accuracy	–	–	90.8% (88.9–92.7%)	92.6% (91.0–94.2%)
Atrial fibrillation	F1 score	0.532 (0.495–0.569)	0.642 (0.611–0.673)	0.783 (0.762–0.804)	0.901 (0.881–0.921)
	Grounding accuracy	–	–	90.0% (88.0–92.0%)	92.3% (90.7–93.9%)
Prior stroke	F1 score	0.577 (0.548–0.606)	0.682 (0.651–0.713)	0.821 (0.801–0.841)	0.941 (0.920–0.962)
	Grounding accuracy	–	–	92.0% (90.3–93.7%)	94.0% (92.5–95.5%)
Narrative (binary)					
Cardiovascular disease	F1 score	0.459 (0.418–0.500)	0.554 (0.521–0.587)	0.721 (0.698–0.744)	0.892 (0.871–0.913)
	Grounding accuracy	–	–	86.5% (84.3–88.7%)	91.2% (89.6–92.8%)
Malignancy	F1 score	0.448 (0.403–0.493)	0.541 (0.508–0.574)	0.712 (0.689–0.735)	0.883 (0.862–0.904)
	Grounding accuracy	–	–	86.0% (83.7–88.3%)	90.8% (89.2–92.4%)
ESRD	F1 score	0.436 (0.382–0.490)	0.532 (0.498–0.566)	0.701 (0.678–0.724)	0.872 (0.851–0.893)
	Grounding accuracy	–	–	85.5% (83.2–87.8%)	90.3% (88.6–92.0%)
Infarction on MRI	F1 score	0.422 (0.370–0.474)	0.523 (0.489–0.557)	0.691 (0.668–0.714)	0.862 (0.841–0.883)
	Grounding accuracy	–	–	85.0% (82.7–87.3%)	89.8% (88.0–91.6%)
Narrative (numerical)					
Initial NIHSS	Mean absolute error	1.998 (1.802–2.194)	1.804 (1.597–2.011)	1.098 (0.894–1.302)	0.853 (0.791–0.915)
	Grounding accuracy	–	–	94.0% (92.6–95.4%)	96.5% (95.3–97.7%)
OCR-based (numerical)					
ASPECT score	Mean absolute error	2.297 (2.102–2.492)	2.003 (1.798–2.208)	1.297 (1.094–1.500)	1.004 (0.941–1.067)
	Grounding accuracy	–	–	92.2% (90.6–93.8%)	95.1% (93.8–96.4%)

RAG, retrieval-augmented generation; HITL, human-in-the-loop; ESRD, end-stage renal disease; MRI, magnetic resonance imaging; NIHSS, National Institutes of Health Stroke Scale; OCR, optical character recognition; ASPECT, Alberta Stroke Program Early CT score

The HITL review encompassed 289 records (24.8% of the total cohort), demonstrating substantial inter-rater agreement between 2 blinded clinicians (Cohen’s κ = 0.89; 95% CI, 0.85 to 0.93). Among these, 93 records were flagged by cosine similarity (58 contained at least one extraction error, 62.4%), 14 by the RAG fallback mechanism (11 contained errors, 78.6%), and 182 were randomly sampled nonflagged records (7 contained errors, 3.8%). This confirmed that automated flagging effectively prioritized records likely to contain errors, while the low error rate in nonflagged records supported the reliability of the automated validation pipeline. Identified error types included ambiguous narrative descriptions (45%), complex temporal expressions (28%), abbreviation ambiguities (18%), and true model hallucinations (9%). Specifically, temporal ambiguity errors frequently arose from expressions such as “hypertension diagnosed several years ago”, where the model struggled to differentiate between active and historical conditions. Abbreviation ambiguities were further exemplified by the term “CA”, which denoted either malignancy or cardiac arrest depending on the specific clinical context, and “CVA”, which sometimes referred to chronic old infarcts rather than acute episodes. Confirmed true hallucinations—defined as extractions entirely unsupported by the source text—were subsequently incorporated into continued LoRA adapter refinement, reducing the hallucination rate from 9% to 2.4% in subsequent test batches, representing a 73% relative reduction and confirming the effectiveness of the iterative HITL feedback loop.

### Stroke outcome prediction performance

**Fig. 4.**
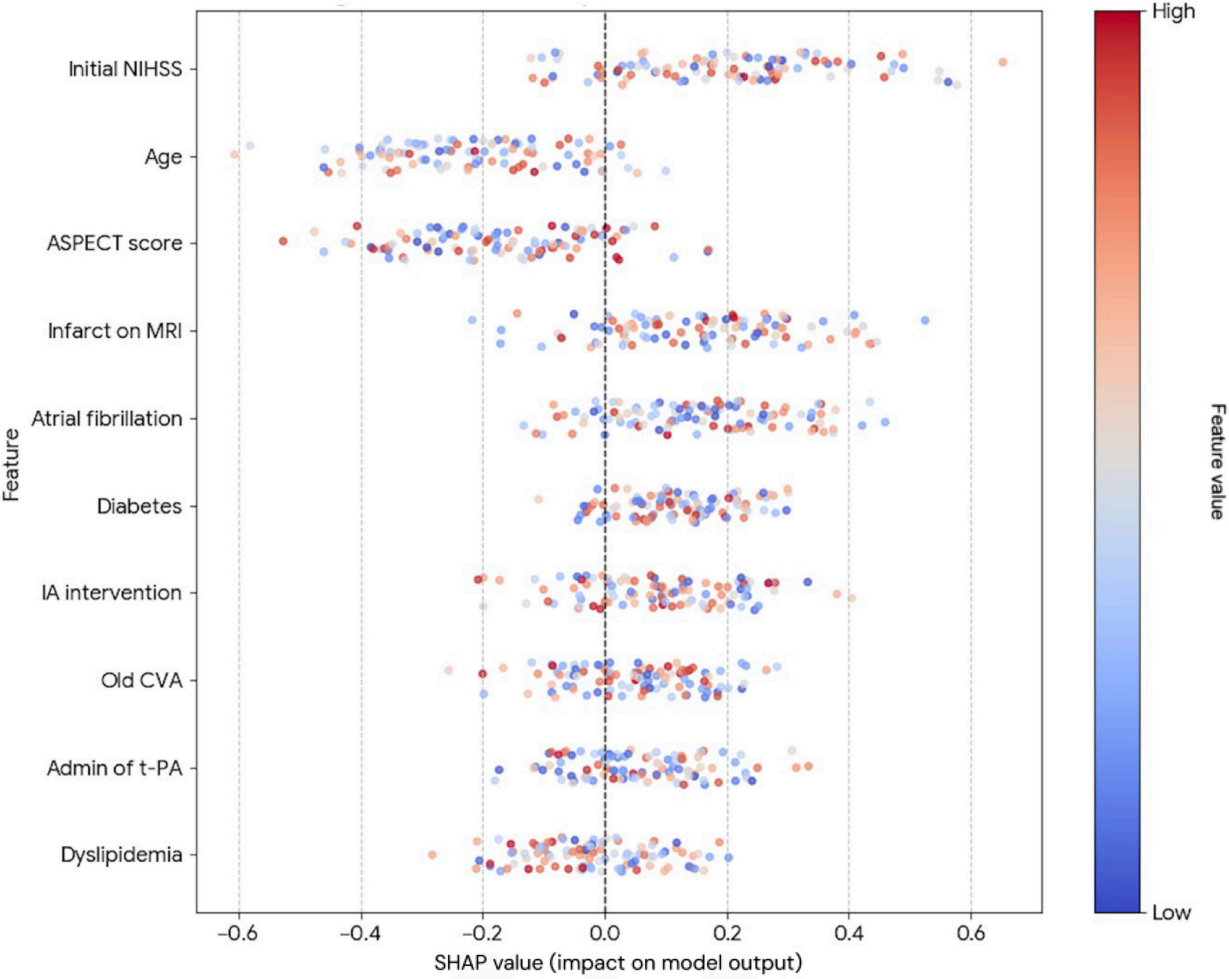
SHAP summary plot for outcome prediction, displaying feature importance of TabPFN model. NIHSS, National Institutes of Health Stroke Scale; ASPECT score, Alberta Stroke Program Early CT score; MRI, magnetic resonance imaging; CVA, cerebrovascular accident; t-PA, tissue plasminogen activator.

The predictive performance of the models on the held-out test set is summarized in Table 3. While all models exhibited acceptable discrimination, the TabPFN model demonstrated the highest AUROC (0.816; 95% CI, 0.784 to 0.847), followed by CatBoost (0.789; 95% CI, 0.756 to 0.822) and logistic regression (0.700; 95% CI, 0.665 to 0.735). In contrast, AUPRC values were similar across all models (~0.315), reflecting the inherent class imbalance of the dataset. All models demonstrated good calibration, passing the Hosmer–Lemeshow goodness-of-fit test (*P* > 0.05). For the best-performing TabPFN model, the Brier score was 0.15 (reference: no-skill model ~0.20) and the expected calibration error (ECE) was 0.05, indicating reliable probabilistic calibration. A calibration curve is presented in Fig. S1. The SHAP value analysis for the TabPFN model is presented in Fig. 4. SHAP analysis of the TabPFN model revealed that higher initial NIHSS, older age, lower ASPECT scores, and the presence of atrial fibrillation or prior stroke were major contributors to poor predicted outcomes. In contrast, t-PA administration and IA intervention were associated with better predicted prognosis, aligning with established clinical evidence.

**Table 3.** Predictive performance of machine learning models on test set, reporting accuracy, precision, recall, F1 score, AUROC, and AUPRC with 95% CIs. All models demonstrated good calibration (Hosmer–Lemeshow *P* > 0.05).

Model	Accuracy (95% CI)	Precision (95% CI)	Recall (95% CI)	F1 score (95% CI)	AUROC (95% CI)	AUPRC (95% CI)
Logistic regression	0.732 (0.685–0.779)	0.511 (0.456–0.566)	0.535 (0.479–0.591)	0.523 (0.468–0.578)	0.700 (0.665–0.735)	0.315 (0.281–0.349)
CatBoost	0.791 (0.748–0.834)	0.583 (0.529–0.637)	0.612 (0.557–0.667)	0.597 (0.543–0.651)	0.789 (0.756–0.822)	0.315 (0.281–0.349)
TabPFN	0.817 (0.776–0.858)	0.615 (0.561–0.669)	0.651 (0.597–0.705)	0.633 (0.579–0.687)	0.816 (0.784–0.847)	0.316 (0.282–0.350)

CI, confidence interval; AUROC, area under the receiver operating characteristic curve; AUPRC, area under the precision–recall curve

The average end-to-end inference time was 8.3 s (range: 7.5 to 9.1 s) per patient record under warm-cache conditions, where model weights are preloaded into memory, reflecting the expected latency in a continuously running departmental system. This performance translates to a throughput of approximately 171 tokens per second, allowing for the processing of comprehensive clinical narratives within seconds.

## Discussion

This proof-of-concept study demonstrates the feasibility of a fully integrated, end-to-end pipeline that transforms unstructured clinical narratives into high-fidelity structured data, subsequently enabling predictive modeling. Utilizing a locally deployed SLM, our pipeline achieves clinically meaningful performance without reliance on cloud infrastructure, thereby preserving data privacy and offering a potentially extensible framework tailored to decentralized clinical settings. Notably, this approach bridges the longstanding gap between narrative data and structured analytics through a multi-layered validation process, achieving extraction accuracy that rivals larger cloud-based models while maintaining compliance with privacy regulations. Importantly, the downstream predictive model, developed solely from automatically extracted variables, underscores the potential of this system to translate raw clinical text into structured data suitable for downstream research, clinical decision support, and quality improvement.

The most clinically significant finding of this study is the demonstration that a locally deployed SLM, when augmented with a structured validation framework, can achieve extraction accuracy sufficient to support downstream predictive modeling without manual curation. The progressive accuracy gains across validation stages reflect the complementary nature of each layer: Whereas rule-based and RAG-based mechanisms addressed errors amenable to automated resolution, the relatively modest proportion requiring human review—approximately 8% to 9% in prospective deployment—suggests that the pipeline strikes a pragmatic balance between automation and oversight. The narrower performance gap between template- and narrative-based variables than might be expected is particularly noteworthy, as it implies that semantic attention mechanisms can partially compensate for the inherent variability of free-text clinical documentation. Most importantly, the fact that predictive performance derived from automatically extracted variables was comparable to benchmarks from manually curated datasets validates the core premise of this work: that automation need not compromise the clinical utility of structured data. Moreover, given that the manual abstraction of comparable clinical variables typically requires 15 to 30 min per case, this sub-10-s processing time is operationally feasible for concurrent clinical use within the 10- to 30-min window of acute stroke triage. This efficiency supports the integration of the pipeline into active clinical workflows without introducing significant latency.

An important finding requiring interpretation is the divergence between high correctness metrics (F1 ~0.92) and moderate semantic similarity scores (BERTScore 0.71, ROUGE-L 0.68). This reflects the fundamental nature of our task: converting narrative text into structured JSON outputs rather than generating semantically similar prose. When a note states “patient has poorly controlled hypertension requiring multiple medications”, our system extracts {“Hypertension”: “yes”}—factually correct but lexically dissimilar. Semantic similarity metrics, designed for generative tasks like summarization, are not well-suited for structured extraction where informational accuracy matters more than textual overlap. This interpretation is supported by the preserved predictive performance; if substantial clinical information were lost, the AUROC would have degraded significantly. This divergence itself highlights a broader methodological consideration: For structured clinical extraction tasks, informational correctness—rather than lexical preservation—is the clinically relevant criterion. This observation contributes a methodological insight for future clinical NLP benchmarking, suggesting that correctness-based metrics should be prioritized over semantic similarity measures when evaluating narrative-to-structured extraction pipelines. Nevertheless, the binary schema discards severity nuances present in narratives, suggesting that hierarchical extraction capturing both presence and granular detail could enhance future iterations [22]. To further contextualize our approach, a qualitative comparison with alternative extraction paradigms is warranted. Rule-based clinical NLP systems have historically achieved F1 scores ranging from 0.70 to 0.90 in well-defined tasks and offer interpretability advantages, but require extensive manual feature engineering and exhibit poor portability across institutions due to sensitivity to documentation style variations [23]. Direct comparisons suggest that while rule-based approaches can be competitive in narrow tasks, they show greater performance degradation than LLM-based systems when applied across heterogeneous documentation styles [24]. Cloud-based LLM approaches such as GPT-4 have demonstrated greater than 85% accuracy in clinical extraction [9], but require transmission of protected health information to external servers. Notably, recent evidence demonstrates that fine-tuned open-source models of comparable scale to our pipeline—such as Llama 3 8B—can achieve human-level extraction performance with minimal training data [25], corroborating the feasibility of our locally deployed approach. Our pipeline thus occupies a pragmatic middle ground: combining the privacy preservation of local deployment with the semantic flexibility of transformer-based models, augmented by a multi-tiered validation framework that systematically mitigates hallucination risk.

The multi-tiered validation framework represents a pragmatic solution to current SLM limitations. Each layer—rule-based verification, RAG-based contextual validation, cosine similarity flagging, and human review—addresses distinct error types, with the defense-in-depth approach achieving statistically significant improvements at each stage (*P* < 0.001 for all comparisons). The rule-based layer rapidly corrected syntactic errors, while RAG cross-verification achieved 93.2% grounding accuracy by reintroducing source text fragments to validate extractions [17]. Cosine similarity flagging identified 8% of records as semantic outliers, of which 62% contained true errors, effectively triaging cases for human review [18]. Crucially, HITL corrections (inter-rater agreement κ = 0.89) were fed back into the model’s LoRA adapters, reducing hallucination rates by 73% in subsequent batches. In prospective clinical deployment, the operational burden of HITL review is expected to be substantially lower than the 24.8% review rate observed in this validation study, which intentionally included a 10% random audit of nonflagged records for quality assurance purposes. Under steady-state deployment, clinician review would be triggered for approximately 8% to 9% of cases flagged by automated mechanisms. Given the pipeline’s 8.3-s inference time and the RAG mechanism’s ability to surface-relevant source sentences directly alongside extracted values, verification can be completed within minutes—particularly since the reviewing clinician has typically just evaluated the patient in question. The domain-specific tuning on neurology department notes enhanced contextual accuracy for institution-specific phrasing [26]. However, this multi-layered approach, while effective, reveals that current SLMs require substantial oversight for clinical deployment—a trade-off between automation and reliability that must be carefully managed in real-world implementation. Under distribution shift to institutions with heterogeneous documentation patterns, the most vulnerable components are the rule-based verification layer, RAG retrieval index, and cosine similarity threshold—all of which are corpus-dependent. The 0.82 similarity threshold, derived empirically from our institutional corpus, would require recalibration in new deployment settings. This concern is supported by recent evidence demonstrating that single-institution clinical artificial intelligence (AI) and NLP models can experience accuracy reductions exceeding 20% when directly applied to external institutions without domain adaptation [27], and that cross-institutional variability in documentation practices, EHR structures, and linguistic patterns represents a well-documented barrier to clinical AI generalizability [28]. Lightweight LoRA-based domain adaptation with a modest set of locally annotated records—as few as 100 to 250 documents have been shown to substantially recover performance [29]—is therefore recommended as part of any cross-institutional deployment strategy.

In the privacy-sensitive field of medicine, where cloud-based models pose significant security risks, our model adopts a privacy-by-design approach [12]. By enabling local deployment, we offer a practical alternative to remote inference models while ensuring compliance with data governance standards. This is particularly relevant for institutions bound by regulations such as Health Insurance Portability and Accountability Act and General Data Protection Regulation, where transmitting protected health information to third-party servers introduces legal and ethical complexities. From a methodological standpoint, our study offers an end-to-end pipeline that consolidates fragmented steps typically examined in isolation—namely, data extraction and predictive modeling. Prior literature often focuses on either one, rarely both within the same clinical study [22,30]. Furthermore, our use of a compact SLM, deployed entirely on-premise, offers a practical counterpoint to cloud-based solutions that, while powerful, pose regulatory and economic challenges. The pipeline’s modular design allows each verification layer to be independently evaluated, tuned, or replaced—providing flexibility for future domain-specific customization [31].

Regarding predictive modeling, the TabPFN model achieved an AUROC of 0.816, suggesting fair-to-good discriminative ability. For context, prior machine learning studies on acute stroke outcome prediction have reported AUROC values ranging from 0.83 to 0.89 using manually curated datasets [19], and a meta-analysis of stroke prognosis models reported a pooled AUROC of 0.79 [20]. Although direct comparison is not appropriate given differences in model architecture, cohort size, and feature curation, our result falls within a clinically comparable range—notably achieved using automatically extracted rather than manually curated variables, underscoring the practical value of the proposed pipeline. This performance, while not exceeding benchmark results from deep learning models trained on manually curated datasets, remains significant [32]. The near-identical AUPRC values across models (~0.315) reflect a structural limitation imposed by the small absolute size of the positive class (*N* ≈ 43 in the test set). Unlike AUROC, which measures relative rank-ordering and is less sensitive to class imbalance, AUPRC is directly constrained by the number of true positives available at high precision thresholds. Consequently, even meaningful improvements in rank discrimination do not translate to observable gains in precision–recall performance under these conditions. The clinical variables used—though extracted with high precision—are relatively standard and do not include novel biomarkers or multimodal features. Nevertheless, the objective was not to establish a new state-of-the-art in stroke prediction, but to validate that data automatically extracted from unstructured text could be directly utilized for clinically relevant modeling. The SHAP value analysis revealed that higher initial NIHSS, older age, lower ASPECT scores, and the presence of atrial fibrillation or prior stroke were major contributors to poor predicted outcomes, while t-PA administration and IA intervention were associated with better predicted prognosis, aligning with established clinical evidence [19,20]. This concordance suggests that the extraction pipeline preserved clinically meaningful relationships despite automation.

Clinically, the system has strong implications for integration into EHRs. By automating structured data entry, it can reduce the burden on clinicians while maintaining or improving documentation quality [31]. More broadly, the approach contributes to the development of a Learning Health System, where data from routine care is transformed into feedback for continuous improvement [33,34]. Our pipeline supports this goal by enabling the conversion of unstructured records into research-grade datasets on a daily basis. However, the integrated pipeline’s viability rests on critical prerequisites. First, extraction accuracy must be exceptionally high to avoid garbage-in-garbage-out effects in downstream models [35]. Second, the system must be capable of reliably identifying all variables required for prediction. In resource-limited settings—such as outpatient clinics, rural hospitals, or specialized departments—the proposed architecture offers substantial advantages, enabling data-driven quality improvement and research cycles without requiring enterprise-level IT infrastructure or third-party data sharing, thereby democratizing evidence generation [36].

Several limitations must be acknowledged. First, the retrospective, single-center design of this study introduces potential for selection and information bias, and the absence of external validation represents an inherent limitation of this proof-of-concept work. Clinical narratives often exhibit significant heterogeneity across different medical centers. Although our SLM-based pipeline is designed to focus on semantic meaning rather than rigid templates to minimize the impact of varied note structures, performance variability remains possible in different clinical settings. Future research involving multi-center validation and lightweight domain adaptation, such as LoRA-based fine-tuning, will be essential to ensure robust performance across diverse healthcare environments. Multicenter validation using federated learning could enhance generalizability without compromising privacy [37]. The application of an English-pretrained SLM to Korean-language text introduces linguistic challenges. While the model’s multilingual capabilities and domain-specific fine-tuning yielded acceptable performance, the higher token-to-character ratio suggests suboptimal tokenization, potentially affecting semantic nuance and increasing computational cost. Future research should pursue Korean-native medical LLMs or continued pretraining on Korean clinical corpora. The black-box nature of LLMs limits interpretability, complicating clinical trust in model decisions. The pipeline’s dependence on historical data also raises the risk of perpetuating embedded biases or outdated practices. From an operational standpoint, automation bias must be mitigated through transparent error reporting and ongoing machine learning operations (MLOps) practices to maintain model integrity as clinical guidelines evolve [38]. Prior to prospective clinical integration, a staged validation process—including shadow mode deployment, prospective accuracy audit, and formal clinical impact assessment—is recommended to ensure patient safety and regulatory compliance. This aligns with emerging regulatory frameworks for clinical AI systems, including the United States Food and Drug Administration’s guidance on AI/ML-based Software as a Medical Device and the European Union Artificial Intelligence Act, both of which emphasize iterative performance monitoring, transparency, and human oversight as prerequisites for safe clinical deployment. Scalability remains another consideration; although optimized for local hardware, increasing data volume could strain available resources. The outcome prediction model employed a dichotomized mRS, potentially oversimplifying the functional recovery spectrum. Future work should explore ordinal regression or fine-grained outcome prediction for more nuanced risk stratification. Integration of multimodal data—such as combining text and imaging features beyond ASPECT scores—would further enrich prediction models. Furthermore, while SMOTE was applied to address class imbalance in the prediction modeling cohort, the clinical plausibility of synthetically generated minority samples—particularly for binary clinical variables—remains a limitation of the current approach. Future implementations should consider constraint-based oversampling methods or, where model architecture permits, class-weighted loss functions as clinically more appropriate alternatives. Additional exploration of natural language instruction tuning and domain-aligned pretraining could refine model performance, particularly in non-English settings.

## Conclusion

This study introduces a proof-of-concept, privacy-conscious pipeline for structured data extraction and outcome prediction within a single-center retrospective setting. By combining a locally deployed SLM with a multi-tiered validation framework, we demonstrate that clinically meaningful extraction accuracy can be achieved on consumer-grade hardware without cloud infrastructure—with the fully automated pipeline achieving substantial accuracy gains through successive validation stages, and the complete human-assisted pipeline reaching 97.0%, reflecting a pragmatic human–machine collaboration model. However, these findings should be interpreted within the constraints of a retrospective, single-institution design; the observed performance may not generalize to institutions with heterogeneous documentation practices or larger data volumes. Prospective multi-center validation is a prerequisite before broader clinical deployment. Despite these constraints, this work establishes a reproducible and privacy-preserving template that may inform future implementation efforts, offering a viable path for healthcare institutions to harness AI capabilities while maintaining data sovereignty.

## Ethical Approval

This retrospective study was approved by the Yonsei University Health System Institutional Review Board (4-2025-0125) and conducted in accordance with the Helsinki Declaration and its later amendments. Informed consent was waived due to the retrospective nature of the study.

## Data Availability

Due to hospital privacy policies and ethical restrictions, the patient data used in this study cannot be publicly shared. Access may be granted upon approval from the institutional review board.

## Supplementary Materials

Supplementary 1 Fig. S1 Code Repository Interactive Demonstration
