# Supplementary material for: End-to-End Pipeline Integrating Local Small Language Models and Machine Learning for Data Extraction and Stroke Outcome Prediction in Emergency Department
Source: Comput Struct Biotechnol J. 2026 Apr 30;35(2):0064. doi: 10.34133/csbj.0064 (PMC13394967; doi:10.34133/csbj.0064)
Supplement: Supplementary 1 — Fig. S1 Code Repository Interactive Demonstration [file csbj.0064.f1.zip › Supplementary Material.docx]

**Supplementary Material**

Supplementary Fig. 1. Calibration curve of the TabPFN model on the held-out test set. Expected Calibration Error (ECE) = 0.05; Brier score = 0.15.

Code Repository: The implementation code is available at https://github.com/Rompy/stroke-outcome-pipeline. The repository contains the core extraction pipeline, multi-tiered validation framework, and prediction model architecture. Note that patient-specific data, fine-tuning datasets, and detailed hyperparameter configurations have been excluded to protect patient privacy and institutional data governance requirements.

Interactive Demonstration: A conceptual demonstration of the pipeline workflow is available at https://stroke-pipeline-demo-rrxshibccyt6dwbsymld9u.streamlit.app. This mock demonstration visualizes the end-to-end data flow from clinical note input through multi-tiered validation to outcome prediction, illustrating the pipeline architecture without utilizing actual patient data or the trained model weights.
