# Supplementary figures and images for: End-to-End Pipeline Integrating Local Small Language Models and Machine Learning for Data Extraction and Stroke Outcome Prediction in Emergency Department
Source: Comput Struct Biotechnol J. 2026 Apr 30;35(2):0064. doi: 10.34133/csbj.0064 (PMC13394967; doi:10.34133/csbj.0064)

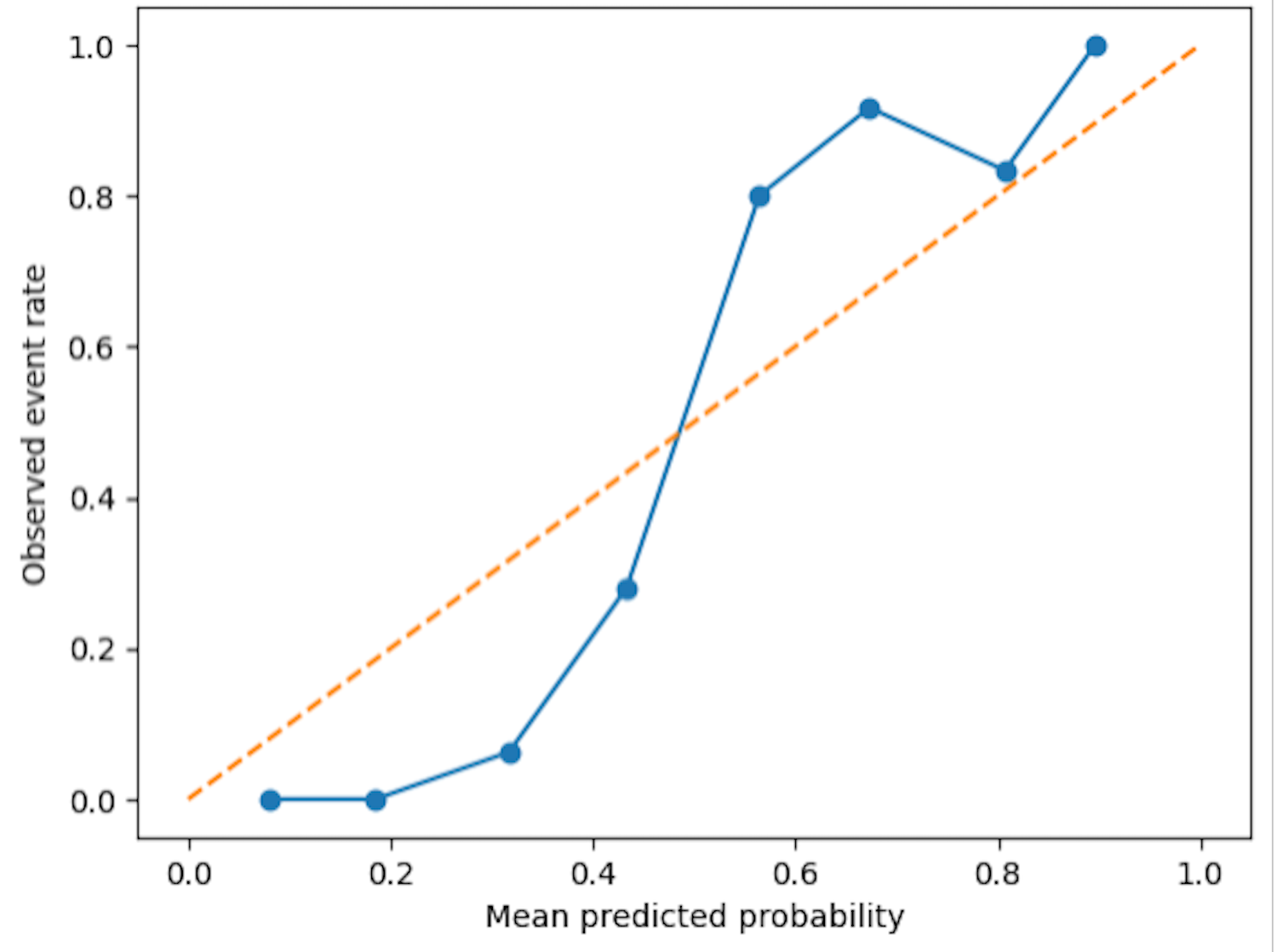

Supplement: Supplementary 1 — Fig. S1 Code Repository Interactive Demonstration [file csbj.0064.f1.zip › Supple Fig1.tiff]
